# Mouse model for chronic liver damage and fibrosis

**Published:** 2014-03

Persistent injury to the liver eventually leads to fibrosis and, in the worst cases, liver failure. Although several rodent models for inducible, acute liver fibrosis are available, only a few transgenic mouse models have been described that spontaneously develop liver fibrosis. Now, Kong et al. have created a novel transgenic mouse model for chronic liver fibrosis by using a gene-trap strategy to disrupt the *Ncu-g1* gene, which encodes a lysosomal membrane protein that is expressed in the liver. *Ncu-g1^gt/gt^* mice develop and grow normally, the researchers report, but spontaneously develop liver fibrosis and exhibit the molecular hallmarks of well-established fibrosis by the age of 6 months. *Ncu-g1^gt/gt^* mice might, therefore, provide an animal model for the study of chronic liver injury and liver fibrosis, and for the development of treatments for these conditions. **Page 351**

